# Identifying cancer biomarkers by network-constrained support vector machines

**DOI:** 10.1186/1752-0509-5-161

**Published:** 2011-10-12

**Authors:** Li Chen, Jianhua Xuan, Rebecca B Riggins, Robert Clarke, Yue Wang

**Affiliations:** 1 Department of Electrical and Computer Engineering, Virginia Polytechnic Institute and State University, Arlington, VA, USA; 2 Departments of Oncology and Physiology & Biophysics, Georgetown University School of Medicine, Washington, DC, USA

## Abstract

**Background:**

One of the major goals in gene and protein expression profiling of cancer is to identify biomarkers and build classification models for prediction of disease prognosis or treatment response. Many traditional statistical methods, based on microarray gene expression data alone and individual genes' discriminatory power, often fail to identify biologically meaningful biomarkers thus resulting in poor prediction performance across data sets. Nonetheless, the variables in multivariable classifiers should synergistically interact to produce more effective classifiers than individual biomarkers.

**Results:**

We developed an integrated approach, namely network-constrained support vector machine (netSVM), for cancer biomarker identification with an improved prediction performance. The netSVM approach is specifically designed for network biomarker identification by integrating gene expression data and protein-protein interaction data. We first evaluated the effectiveness of netSVM using simulation studies, demonstrating its improved performance over state-of-the-art network-based methods and gene-based methods for network biomarker identification. We then applied the netSVM approach to two breast cancer data sets to identify prognostic signatures for prediction of breast cancer metastasis. The experimental results show that: (1) network biomarkers identified by netSVM are highly enriched in biological pathways associated with cancer progression; (2) prediction performance is much improved when tested across different data sets. Specifically, many genes related to apoptosis, cell cycle, and cell proliferation, which are hallmark signatures of breast cancer metastasis, were identified by the netSVM approach. More importantly, several novel hub genes, biologically important with many interactions in PPI network but often showing little change in expression as compared with their downstream genes, were also identified as network biomarkers; the genes were enriched in signaling pathways such as TGF-beta signaling pathway, MAPK signaling pathway, and JAK-STAT signaling pathway. These signaling pathways may provide new insight to the underlying mechanism of breast cancer metastasis.

**Conclusions:**

We have developed a network-based approach for cancer biomarker identification, netSVM, resulting in an improved prediction performance with network biomarkers. We have applied the netSVM approach to breast cancer gene expression data to predict metastasis in patients. Network biomarkers identified by netSVM reveal potential signaling pathways associated with breast cancer metastasis, and help improve the prediction performance across independent data sets.

## Background

While promising progress in research has been made in recent years, predicting cancer outcomes is a difficult task since cancer is a complicated disease and its mechanisms remain largely unclear. Biomarkers play an important role in the diagnosis of cancer, and also in assessing prognosis and directing treatment of cancer. As microarray technology makes it possible to measure the expression of tens of thousands of genes simultaneously, biomarker identification has become one of the major tasks in the field of microarray data analysis. Common statistical practice attempts to find biomarkers differentially expressed across different phenotypes, such as cancer samples vs. normal samples, in a high-dimensional gene space. Given clinical outcomes data, the problem can also be formulated as a prediction problem that is designed to find informative genes from a classification model with good prediction performance.

Traditional methods [1-8] are largely developed based on microarray data alone, with the assumption that each individual gene contributes independently to clinical outcomes. Thus, the reproducibility of prediction performance is often unexpectedly low when tested across different data sets (even though data are acquired from apparently similar study designs). This problem may be explained in part by the properties of microarray data that are often noisy and the cellular and molecular heterogeneity of cancer specimens. Unfortunately, biomarkers selected by many current algorithms often have limited mechanistic coherence related to the specific cancer under study, partly because the approaches do not deal effectively with the challenges posed by working in high dimensional data spaces [9].

Genes generally work collaboratively, and many cancer-related genes are involved in multiple pathways [10]. Recently, several methods have been developed to identify significant gene sets or pathways involved in diseases or biological processes by incorporating some prior biological knowledge. For example, gene set enrichment analysis or pathway enrichment analysis [11-13] uses the membership information in functional gene clusters or pathways, which facilitates an understanding of the underlying biological mechanism(s). Other algorithms use interacting structures, such as protein-protein interactions (PPIs), protein-DNA interactions, or regulatory pathways. For example, Chuang *et al*. [14] proposed a protein-protein network-based approach to identify biomarkers of metastasis using breast cancer gene expression data. Biomarkers identified by this approach are encoded as subnetworks of interacting proteins within a large human PPI network. The average expression activities of these subnetworks were then used for prediction of metastasis. A noticeable limitation of this method is that the network structure was not taken into consideration in the classifier building step. Li *et al*. [15] introduced a network-constrained regularization procedure for linear regression analysis of microarray data. Specifically, a network-constrained term based on the L1-norm of regression coefficients was used to enforce the smoothness of the coefficients for each network. In a general regression framework, the effectiveness of this approach has been initially demonstrated with relevant genes or subnetworks identified showing an improved association with the appropriate phenotypes. However, in many cases only binary information of clinical outcomes are known (recurrent/non-recurrent, alive/dead), therefore a binary prediction model is more suitable than a regression model for cancer prediction. Zhu *et al*. [16] recently started using support vector machines to build binary classifiers as prediction models, in which an F_∞_-norm constraint was proposed to account for gene-gene interaction information. As an initial attempt, they applied this approach to breast cancer data to study three small, focused networks centered upon TP53, BRCA1, and BRCA2, respectively, showing the potential of this approach to identify those frequently mutated cancer related genes, although the results apply to genes largely known from previous studies [16].

We have developed an integrated approach, network-constrained support vector machine (netSVM), to predict clinical outcome of patients and to identify biologically meaningful biomarkers by incorporating protein-protein interacting network information. Specifically, we embed a network constraint into the objective function of an SVM to impose the smoothness of coefficient over a prediction network. The network constraint is represented by a Laplacian matrix of protein-protein interactions. We first validate the netSVM approach using simulation studies to explore the effectiveness of the proposed method. We then apply the netSVM to breast cancer data for cancer biomarker identification. The study shows that our method can be used to improve the prediction performance across data sets, especially when signal-to-noise ratio (SNR) is relatively low. More importantly, the identified genes and subnetwork are highly related to biological pathways involved in breast cancer progression and metastasis.

## Results and discussion

### Network-constrained support vector machines

**Figure 1:**
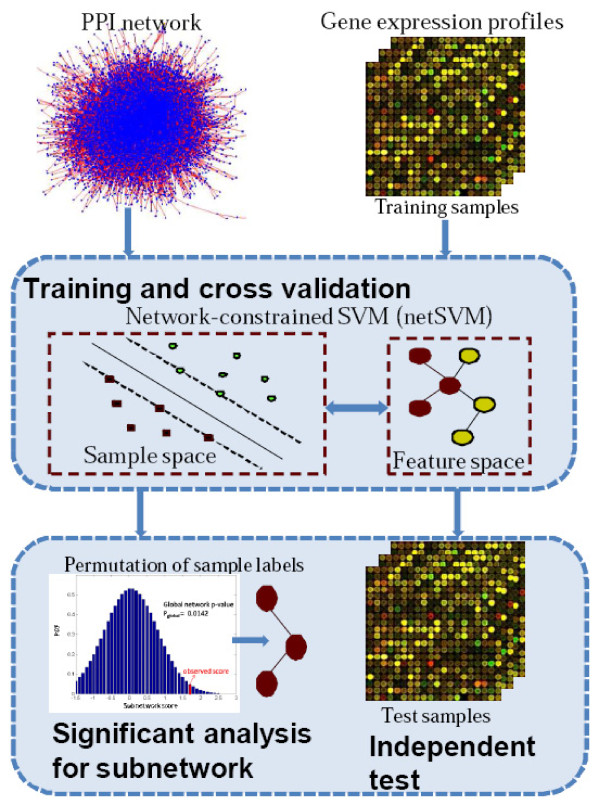
**Flowchart of the proposed approach, network-constrained support vector machine (netSVM), for cancer biomarker identification**.

We propose an integrated approach using gene expression data and PPI network information to predict clinical outcomes of breast cancer and to identify cancer biomarkers. For these studies, we are less interested in describing clinically useful classifiers than we are in using clinically relevant outcomes data to support a classifier from which we can obtain mechanistically relevant biological insights. Figure 1 shows the framework of the proposed method. The method takes gene expression data and PPI network knowledge as the input, builds a classifier using a network-constrained support vector machine (netSVM), and then predicts the outcome of new samples based on the trained classifier. Significant genes or subnetworks from the classifier can be detected through a significance test based on permutation of sample labels. Unlike conventional SVM, netSVM adds a network constraint in the gene space to its objective function; thus we obtain highly connected genes as the significant features and should improve prediction performance across different data sets. The approach is described in the Methods part with its mathematical details outlined.

### Simulation experiments

We simulated microarray gene expression data under two conditions by a modified MRF-GG model [17]. First, a Markov random field (MRF) model was used to determine the states of genes - differentially expressed (DE) and equally expressed (EE) - given a known, ground truth subnetwork. Based on the states of genes, the Gamma-Gamma (GG) model [18] was then used for modeling the gene expression levels in the two conditions (see Methods).

We conducted simulation studies on a breast cancer-related network that contains 584 genes and 2,280 interactions. Genes are either breast cancer related [19] or involved in estrogen signaling pathways collected from Ingenuity Pathway Analysis (Ingenuity^® ^Systems, http://www.ingenuity.com). Interactions were extracted from the HPRD database [20]. Weights in the network were set to 1 if there are known connections between two genes, and to 0 otherwise. Parameters in the GG model (*α *= 10, *α_0 _*= 0.9 and *ν *= 0.5) are those in Newton *et al*. [18]. When generating simulation data sets, we added different levels of noise and adjusted parameter *w *(see Eq. 11 in Methods) to control the false positive rate in the sampled DE subnetworks. For each scenario, we randomly generated 100 training and testing data sets, each data set with 100 training samples and 100 testing samples.

We implemented network-constrained SVMs for training and testing. A 10-fold cross validation was conducted on the training data set to select the optimal value of parameter *λ*, a trade-off parameter between classification error and network constraint (see Eq. 4 in Methods). We then computed the accuracy, sensitivity, and specificity for classification performance evaluation on the testing data. The classifier's performance in recovering ground truth subnetwork genes was also assessed using receiver operating characteristic (ROC) analysis [2] of the ranked gene list. Specifically, genes were ranked by their p-values through a significance test. True positive rate and false positive rate were calculated in the ranked gene list, and the area under the ROC curve (AUC) were calculated for an overall performance evaluation.

As a comparison, we also implemented many existing methods for classifier training and performance evaluation. Among them, F_∞_-norm SVM [16], Larsnet [15] and Chuang's method [14] are network-based methods that integrate gene expression data and protein-protein interaction network information. Conventional SVM, Lasso [21] and Linear Discriminant Analysis (LDA) [22] are gene-based methods that are based on gene expression data alone. Note that for LDA we used t-test to select top ranked (significant) genes for prediction if number of genes is greater than number of samples. Similarly, we conducted 10-fold cross validation to determine the optimal parameters for the methods and compared the classifier's performance in term of prediction accuracy in the outcome of testing samples and recovering ground truth subnetwork genes.

**Figure 2:**
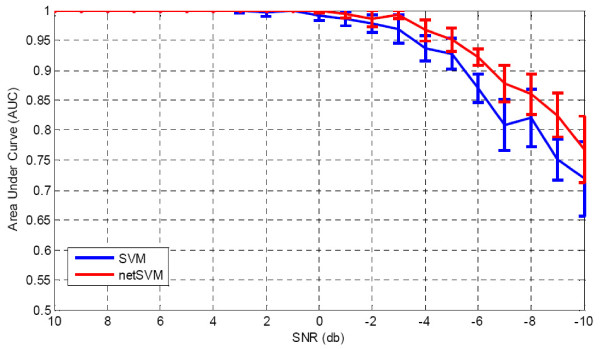
**Comparison results of prediction performance**: AUC values on simulation data sets with different signal-to-noise levels for network-constrained SVM and conventional SVM. AUC values are calculated based on 100 simulations, where standard deviations are shown in the error bars.

**Figure 3:**
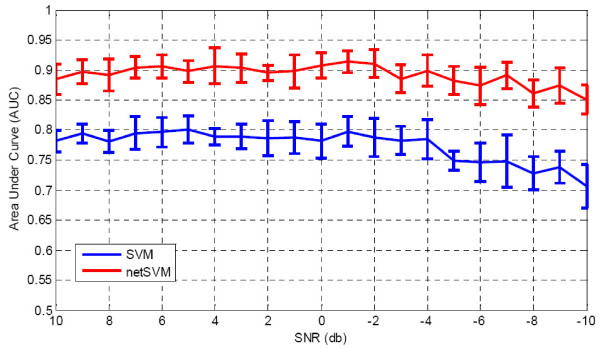
**Comparison results of subnetwork identification**: AUC values on simulation data sets with different signal-to-noise levels for network-constrained SVM and conventional SVM. AUC values are calculated based on 100 simulations, where standard deviations are shown in error bars.

We first fixed weight (*ω *= 10) and added different levels of Gaussian noise to the simulated gene expression data. Table 1 shows that the AUC values of prediction performance on testing data sets for netSVM and other existing methods. From the table we can see that when signal-to-noise ratio is relatively high (>4 db), most of the methods can achieve good prediction results, except for two regression methods, Larsnet and Lasso. However, when signal-to-noise ratio is low, which is a common problem with microarray gene expression data, netSVM gives rise to an improved classification performance compared to other methods. The regression methods do not show good prediction performances in noisy conditions. One possible reason is that the simulation data are generated based on statistical distributions rather than precise regression models. The AUC values for subnetwork identification are shown in Table 2. We can see that network-based methods outperform gene-based methods consistently, and netSVM outperforms all other methods. This indicates that integrating PPI network information could improve discovering underlying subnetworks. Figure 2 and Figure 3 show the detailed comparison between netSVM and conventional SVM in terms of AUC values of prediction performance and subnetwork identification, respectively. NetSVM outperforms SVM significantly in identifying the ground truth subnetwork or relevant genes.

**Table 1:** Means and standard derivations of AUC values of prediction on simulation data sets with different signal-to-noise levels for netSVM, other network-based methods and gene-based methods.

SNR (db)	network-based method			gene-based method		
	
	netSVM	F_∞_-norm SVM	Larsnet	SVM	Lasso	LDA
10	1.00 ± 0.00	1.00 ± 0.00	0.62 ± 0.07	1.00 ± 0.00	0.61 ± 0.04	1.00 ± 0.00
8	1.00 ± 0.00	1.00 ± 0.00	0.60 ± 0.05	1.00 ± 0.00	0.59 ± 0.05	1.00 ± 0.00
6	1.00 ± 0.00	1.00 ± 0.00	0.58 ± 0.05	1.00 ± 0.00	0.57 ± 0.05	1.00 ± 0.00
4	1.00 ± 0.00	1.00 ± 0.00	0.57 ± 0.05	1.00 ± 0.00	0.56 ± 0.03	1.00 ± 0.00
2	1.00 ± 0.00	1.00 ± 0.00	0.57 ± 0.03	1.00 ± 0.00	0.55 ± 0.04	1.00 ± 0.00
0	1.00 ± 0.00	1.00 ± 0.00	0.58 ± 0.03	1.00 ± 0.00	0.57 ± 0.04	0.99 ± 0.01
-2	0.99 ± 0.01	0.98 ± 0.01	0.57 ± 0.03	0.99 ± 0.01	0.56 ± 0.04	0.98 ± 0.01
-4	0.99 ± 0.01	0.94 ± 0.03	0.58 ± 0.04	0.97 ± 0.02	0.57 ± 0.04	0.93 ± 0.01
-6	0.95 ± 0.02	0.88 ± 0.04	0.58 ± 0.03	0.93 ± 0.03	0.56 ± 0.04	0.88 ± 0.03
-8	0.88 ± 0.03	0.81 ± 0.05	0.57 ± 0.04	0.81 ± 0.04	0.56 ± 0.04	0.83 ± 0.02
-10	0.82 ± 0.04	0.73 ± 0.05	0.56 ± 0.04	0.75 ± 0.03	0.57 ± 0.05	0.76 ± 0.02

**Table 2:** Means and standard derivations of AUC values of prediction of subnetwork genes on simulation data sets with different signal-to-noise levels for netSVM, other network-based methods and gene-based methods.

SNR (db)	network-based method				gene-based method		
	
	netSVM	F_∞_-norm SVM	Larsnet	Chuang's method	SVM	Lasso	T-test
10	0.89 ± 0.00	0.80 ± 0.03	0.64 ± 0.02	0.85 ± 0.03	0.79 ± 0.00	0.62 ± 0.02	0.78 ± 0.03
8	0.90 ± 0.02	0.81 ± 0.03	0.64 ± 0.02	0.81 ± 0.03	0.79 ± 0.02	0.62 ± 0.01	0.78 ± 0.04
6	0.90 ± 0.02	0.81 ± 0.03	0.63 ± 0.02	0.84 ± 0.03	0.79 ± 0.03	0.62 ± 0.02	0.77 ± 0.04
4	0.90 ± 0.02	0.81 ± 0.04	0.63 ± 0.01	0.82 ± 0.02	0.80 ± 0.02	0.61 ± 0.01	0.78 ± 0.04
2	0.90 ± 0.02	0.80 ± 0.03	0.63 ± 0.01	0.83 ± 0.02	0.79 ± 0.02	0.62 ± 0.02	0.77 ± 0.04
0	0.90 ± 0.03	0.81 ± 0.03	0.63 ± 0.02	0.83 ± 0.04	0.79 ± 0.03	0.61 ± 0.02	0.78 ± 0.04
-2	0.91 ± 0.02	0.80 ± 0.03	0.63 ± 0.02	0.82 ± 0.03	0.80 ± 0.02	0.61 ± 0.02	0.79 ± 0.03
-4	0.89 ± 0.02	0.79 ± 0.03	0.63 ± 0.01	0.83 ± 0.02	0.78 ± 0.02	0.61 ± 0.02	0.78 ± 0.03
-6	0.88 ± 0.02	0.79 ± 0.03	0.63 ± 0.02	0.83 ± 0.04	0.75 ± 0.02	0.61 ± 0.01	0.76 ± 0.05
-8	0.89 ± 0.02	0.77 ± 0.03	0.63 ± 0.01	0.83 ± 0.03	0.75 ± 0.04	0.61 ± 0.01	0.77 ± 0.04
-10	0.87 ± 0.03	0.75 ± 0.03	0.63 ± 0.02	0.80 ± 0.04	0.74 ± 0.03	0.61 ± 0.01	0.76 ± 0.04

**Figure 4:**
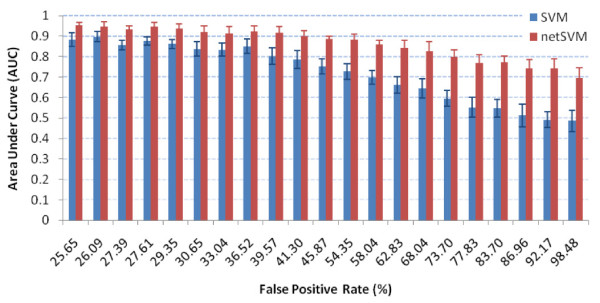
**Comparison results of subnetwork identification**: AUC values on simulation data sets with different false positive rates for network-constrained SVM and conventional SVM. AUC values are calculated based on 100 simulations, where standard deviations are shown in error bars.

We further evaluated the performance of uncovering underlying network/genes with different false positive rates in the data by varying weights (*ω*), to control the false positive rate of sampled subnetworks compared with the ground truth subnetwork. With a fixed signal-to-noise ratio (SNR = 0 dB), the prediction performance of six methods are similar with the ones in Table 1 (results are not shown). However, the performance in identifying underlying subnetworks is substantially different, which is shown in Table 3. From the table, we can conclude that network-based methods outperform gene-based methods in general. Figure 4 shows the detailed comparison between netSVM and conventional SVM. From the figure we can see that netSVM achieves higher AUC values than conventional SVM significantly, especially when false positive rate of sampled subnetwork is high (>40%).

**Table 3:** Means and standard derivations of AUC values of prediction of subnetwork genes on simulation data sets with different false positive rate (FPR) for netSVM, other network-based methods and gene-based methods.

FPR (%)	network-based method				gene-based method		
	
	netSVM	F-norm SVM	Larsnet*	Chuang's method	SVM	Lasso*	T-test
25	0.95 ± 0.02	0.90 ± 0.03	0.93 ± 0.02	0.92 ± 0.03	0.90 ± 0.03	0.81 ± 0.04	0.91 ± 0.02
27	0.95 ± 0.02	0.90 ± 0.03	0.92 ± 0.02	0.93 ± 0.02	0.88 ± 0.02	0.80 ± 0.03	0.89 ± 0.02
29	0.94 ± 0.02	0.89 ± 0.03	0.91 ± 0.02	0.90 ± 0.02	0.86 ± 0.02	0.80 ± 0.03	0.88 ± 0.03
33	0.91 ± 0.03	0.87 ± 0.02	0.89 ± 0.02	0.90 ± 0.02	0.83 ± 0.03	0.79 ± 0.03	0.86 ± 0.04
39	0.92 ± 0.03	0.85 ± 0.03	0.87 ± 0.03	0.87 ± 0.03	0.80 ± 0.04	0.78 ± 0.03	0.83 ± 0.03
46	0.89 ± 0.01	0.83 ± 0.03	0.82 ± 0.02	0.83 ± 0.02	0.75 ± 0.04	0.76 ± 0.03	0.77 ± 0.03
58	0.86 ± 0.02	0.79 ± 0.03	0.77 ± 0.02	0.80 ± 0.02	0.70 ± 0.03	0.72 ± 0.03	0.73 ± 0.04
68	0.83 ± 0.05	0.76 ± 0.03	0.73 ± 0.02	0.72 ± 0.03	0.64 ± 0.05	0.71 ± 0.02	0.66 ± 0.04
77	0.77 ± 0.04	0.70 ± 0.04	0.69 ± 0.02	0.67 ± 0.04	0.55 ± 0.05	0.67 ± 0.02	0.58 ± 0.03
86	0.74 ± 0.04	0.66 ± 0.04	0.64 ± 0.02	0.66 ± 0.03	0.51 ± 0.06	0.63 ± 0.02	0.52 ± 0.04
98	0.70 ± 0.05	0.62 ± 0.04	0.59 ± 0.00	0.56 ± 0.03	0.49 ± 0.05	0.59 ± 0.01	0.47 ± 0.04

**Figure 5:**
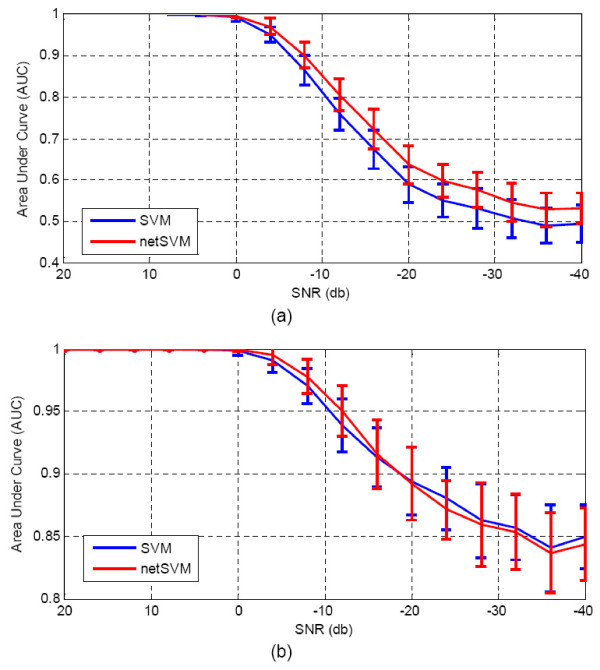
**Comparison results on the robustness of methods**: AUC values on simulation data sets with different signal-to-noise levels for network-constrained SVM and conventional SVM. AUC values are calculated based on 100 simulations, where standard deviations are shown in the error bars. (a) perturbing all ground truth genes in testing data sets; (b) perturbing hub genes (of node degree > 10) in testing data sets.

*: noise = 0; others: SNR = 0 db;

Since our method is designed to emphasize the role of hub genes, the negative effect on prediction accuracy of hub genes is greater than other genes when the genes have inconsistent, abnormal expression between training and testing data sets. We assessed the robustness of the method by perturbing the expression levels of all ground truth genes and hub genes, respectively. Genes were considered as hub genes if their connection degrees are larger than 10. We added different levels of noise in the test data sets and compared the prediction performance of netSVM and that of conventional SVM. From simulation experiments, we can see that netSVM is more robust than conventional SVM when perturbing all ground truth genes (Figure 5(a)). The performance degrades even faster when perturbing hub genes alone, but it is still acceptable when compared to the performance of conventional SVM (Figure 5(b)).

### Breast cancer microarray data

We studied two gene expression profiles of breast cancer patients previously reported by van de Vijver *et al*. [23] and Wang *et al*. [24]. We focused on estrogen receptor (ER) positive patients in our study, aiming to improve our understanding of estrogen signaling and action. Among the ER positive patients, 78 patients in van de Vijver *et al*. [23] and 80 in Wang *et al*. [24] had been diagnosed with metastasis during their follow-up visits within 5 years of surgery, which were assigned to 'recurrence' group.

The remaining 217 and 129 patients, respectively in the two studies, were then labeled as 'non-recurrence'. In order to construct a network, we collected a set of genes that are either breast cancer related [19] or involved in estrogen signaling pathways from Ingenuity Pathway Analysis (Ingenuity^® ^Systems, http://www.ingenuity.com). The protein-protein interactions (PPIs) were extracted from the HPRD database [20]. In this study, the weights in the network are set as 1 if there are connections between two genes and 0 otherwise. After mapping the network to the two gene expression data sets [23,24], we obtained a PPI network with 553 breast cancer related genes and 2,257 interactions.

We conducted a stratified, 5-fold cross validation on the first data set to build a classifier, and then tested on the second data set to measure its prediction performance, and vice versa. For cross-validation with network-constrained SVM, the samples are divided into five subsets: three are used to train the classifier, one is used to determine the optimal value of parameter *λ*, and validation performance is calculated on the remaining subset using the optimal *λ*. To obtain a more reliable evaluation of the performance, we repeated the cross-validation procedure 100 times by random partitions. The most frequently occurring value of parameter *λ *during the cross validation was used to build a classifier based on all training samples for independent testing. We evaluated the prediction performance of netSVM using ROC analysis, from which AUC, accuracy, sensitivity, and specificity were calculated.

**Figure 6:**
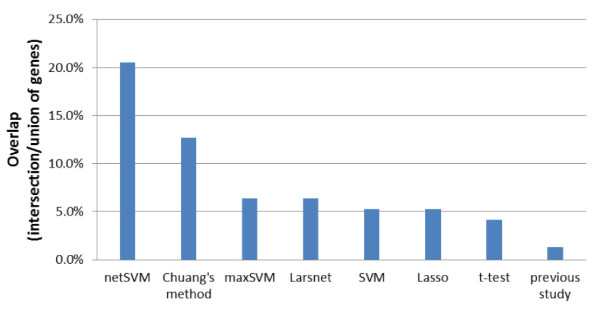
**Overlaps of the top 50 genes selected from van de Vijver *et al*. **[23]**and Wang *et al*. **[24]**using different methods**. The overlap is defined as the number of interaction genes divided by the number of union genes in two gene lists.

Similarly we compared the prediction performance of netSVM with other network-based methods and gene-based methods in terms of cross-validation and independent testing. For the cross-validation performance, Table 4 shows the mean and standard deviation of prediction performance for all methods; network-based methods achieved a slightly better classification performance than the gene-based methods. Table 5 shows the prediction performance of independent testing on two data sets. The prediction performance of netSVM and Chuang's method are comparable and better than other network-based and gene-based methods. This indicates that netSVM, along with Chuang method, is of a better reproducibility to predict independent data sets as compared to conventional SVM and other methods. The overlaps in the top 50 ranked genes from two data sets also show that netSVM has a better reproducibility for network identification (Figure 6).

**Table 4:** Means and standard derivations of AUC, accuracy (ACC), sensitivity (SEN) and specificity (SPE) for 5-fold cross validation on van de Vijver *et al*. [23] (top) and Wang *et al *[24] (bottom) for netSVM, other network-based methods and gene-based methods.

	network-based method				gene-based method			previous study	
	
	net SVM	F_∞_-norm SVM	Larsnet	Chuang's method*	SVM	Lasso	t-test + LDA	70 genes [23]*	76 genes [24]*
AUC	0.68 ±	0.60 ±	0.69 ±	-	0.63 ±	0.61 ±	0.64 ±	-	-
	0.02	0.02	0.02		0.02	0.02	0.02		
ACC	0.65 ±	0.62 ±	0.67 ±	0.70	0.63 ±	0.53 ±	0.64 ±	0.62	-
	0.02	0.02	0.02		0.02	0.02	0.02		
SEN	0.51 ±	0.50 ±	0.71 ±	0.90	0.49 ±	0.73 ±	0.44 ±	0.93	-
	0.04	0.02	0.03		0.04	0.02	0.02		
SPE	0.74 ±	0.67 ±	0.67 ±	0.63	0.71 ±	0.47 ±	0.71 ±	0.53	-
	0.03	0.02	0.02		0.03	0.02	0.02		

AUC	0.73 ±	0.64 ±	0.70 ±	-	0.72 ±	0.68 ±	0.60 ±	-	-
	0.02	0.01	0.02		0.02	0.02	0.02		
ACC	0.71 ±	0.63 ±	0.68 ±	0.72	0.70 ±	0.68 ±	0.59 ±	-	0.62
	0.02	0.01	0.02		0.02	0.02	0.02		
SEN	0.42 ±	0.61 ±	0.65 ±	0.90	0.42 ±	0.63 ±	0.49 ±	-	0.93
	0.04	0.02	0.03		0.04	0.03	0.02		
SPE	0.81 ±	0.64 ±	0.71 ±	0.62	0.80 ±	0.72 ±	0.65 ±	-	0.53
	0.02	0.02	0.02		0.02	0.02	0.02		

*: results are extracted from the study [14].

**Table 5:** AUC, accuracy (ACC), sensitivity (SEN) and specificity (SPE) for independent testing on van de Vijver *et al*. [23] (top) and Wang *et al*. [24] (bottom) for netSVM, other network-based methods and gene-based methods.

	network-based method				gene-based method			previous study	
	
	net SVM	F_∞_-norm SVM	Larsnet	Chuang's method*	SVM	Lasso	t-test + LDA	**70 genes **[23]*	**76 genes **[24]*
AUC	0.61	0.50	0.58	0.72	0.55	0.51	0.54	-	0.50
ACC	0.67	0.64	0.70	0.56	0.62	0.66	0.62	-	0.49
SEN	0.47	0.36	0.38	0.90	0.47	0.33	0.62	-	0.37
SPE	0.75	0.75	0.82	0.43	0.68	0.78	0.62	-	0.54

AUC	0.64	0.60	0.66	0.63	0.62	0.61	0.60	0.60	-
ACC	0.65	0.59	0.60	0.49	0.64	0.62	0.57	0.59	-
SEN	0.53	0.63	0.47	0.90	0.51	0.60	0.44	0.45	-
SPE	0.73	0.58	0.82	0.24	0.72	0.65	0.66	0.67	-

*: results are extracted from the study [14].

We also compared the prediction performance with the performances reported in the original studies [23,24]. 70 gene signatures were identified in van de Vijver *et al*. [23] and 76 gene signatures in Wang *et al*. [24]. In the setting of cross validation netSVM achieved a slightly better prediction performance than the original studies (Table 4). However, in the setting of independent testing across data sets, netSVM achieved a significant improvement in prediction accuracy as compared to that from the 70 or 76 gene signatures identified in the original studies (Table 5). Furthermore, netSVM can identify more overlapped genes from two data sets (~20%) than those of previous studies (~1%) (Figure 6), which indicates that netSVM has a better reproducibility across data sets in terms of prediction performance and biomarker identification.

**Figure 7:**
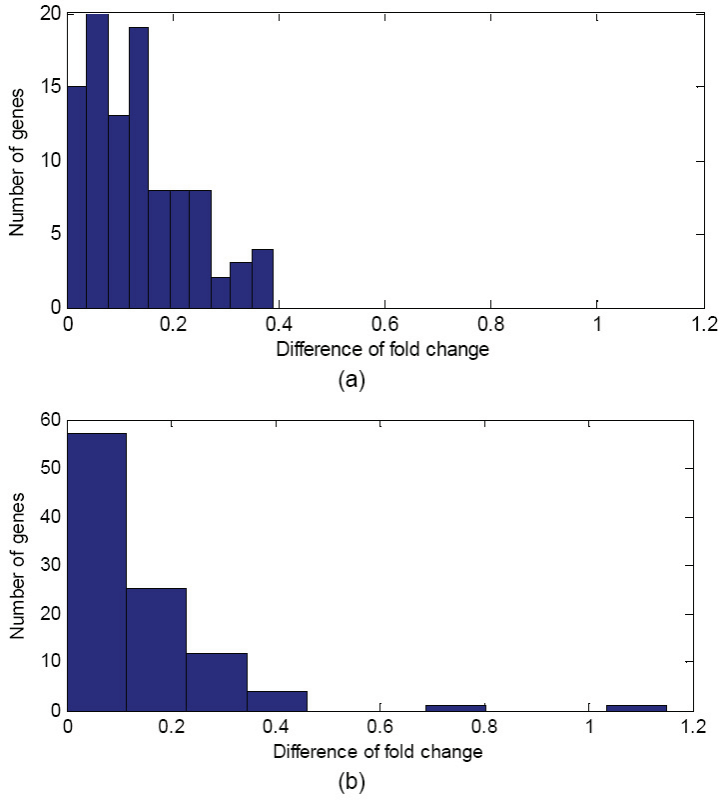
**Histograms of difference of fold change between van de Vijver *et al*. **[23]**and Wang *et al*. **[24]**for (a) hub genes and (b) non-hub genes, respectively**.

As observed in the simulation study, netSVM is more sensitive to hub genes if they have abnormal expression between two data sets. To study the possibility of this effect, we examined the expression changes of hub genes and non-hub genes in two breast cancer data sets. Figure 7 shows the distribution of difference of fold changes between two data sets for 100 hub genes and 100 non-hub genes. The variance of hub genes is in overall smaller than non-hub genes. This observation is consistent with our assumption that hub genes have little expression changes between difference phenotypes, so that they have less variations across different data sets as compared to their down-stream genes. We also conducted a statistical analysis to assess the significance of robustness of selected genes across two data sets. We take the variance of difference of fold change as the summary statistic and generate the null distribution from randomly selected genes (of the same number as the identified genes). The empirical p-value is then calculated by the frequency of occurrences of null variance less than the observed one. The p-values for the top 50 genes selected by netSVM are 0.09 in van de Vijver *et al*. [23] and 0.02 in Wang *et al*. [24], respectively, which are much more significant than those from the genes selected by SVM (0.13 in van de Vijver *et al*. [23] and 0.18 in Wang *et al*. [24], respectively). These results further support and validate that network-based methods can perform better than single gene-based methods.

**Figure 8:**
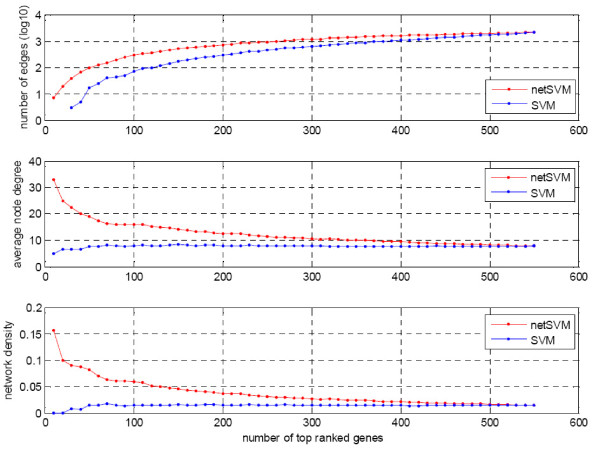
**Comparison of network properties of the top ranked genes identified from van de Vijver *et al*. **[23]**by netSVM and SVM, respectively**.

**Figure 9:**
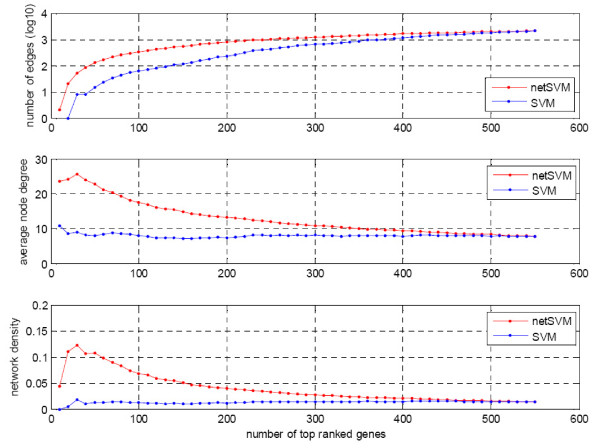
**Comparison of network properties of the top ranked genes identified from Wang *et al*. **[24]**by netSVM and SVM, respectively**.

**Figure 10:**
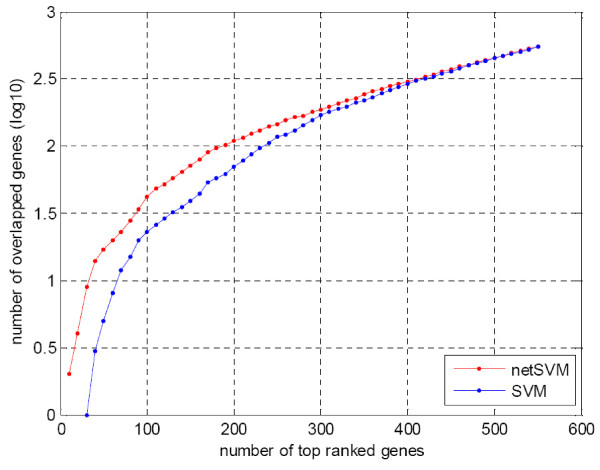
**Number of overlapped genes in the top ranked subnetworks identified from van de Vijver *et al*. **[23]**and Wang *et al*. **[24]**by netSVM and SVM, respectively**.

We further examined the top ranked genes and their composed networks from the classifiers defined by network-constrained SVM and conventional SVM on two data sets. The genes were ranked by their p-values through a significance test (see the Methods section for the detailed procedure). We compared various network properties including number of edges, average node degree and network density. Network density is defined as 2 × *m*/*n*× (*n*-1), where *m *is the number of edges and *n *is the number of nodes in the network. Figure 8 and Figure 9 show the trends of network properties with different network sizes for netSVM and SVM, respectively. From the figures we can see that netSVM results in much denser subnetworks than does SVM for the top ranked genes. Figure 10 shows the number of overlapped genes in the top ranked genes from van de Vijver *et al*. [23] and Wang *et al*. [24]. netSVM results in more overlapped genes in the top ranked subnetworks than SVM, indicating that a good reproducibility can be obtained by using netSVM across different data sets.

**Figure 11:**
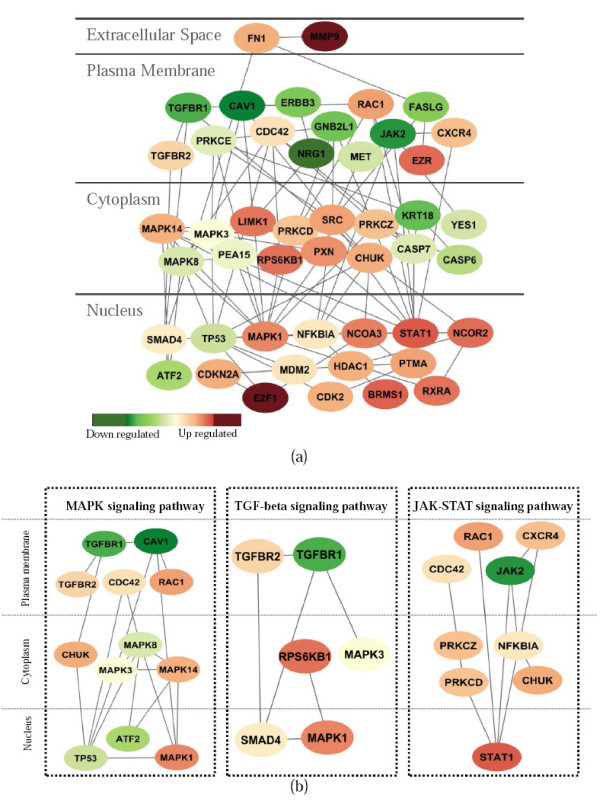
**(a) Subnetworks from the top 50 genes identified by netSVM on van de Vijver *et al*. **[23]**; (b) signaling pathways highlighted in the identified subnetworks including MAPK, TGF-beta and JAK-STAT signaling pathways**.

**Figure 12:**
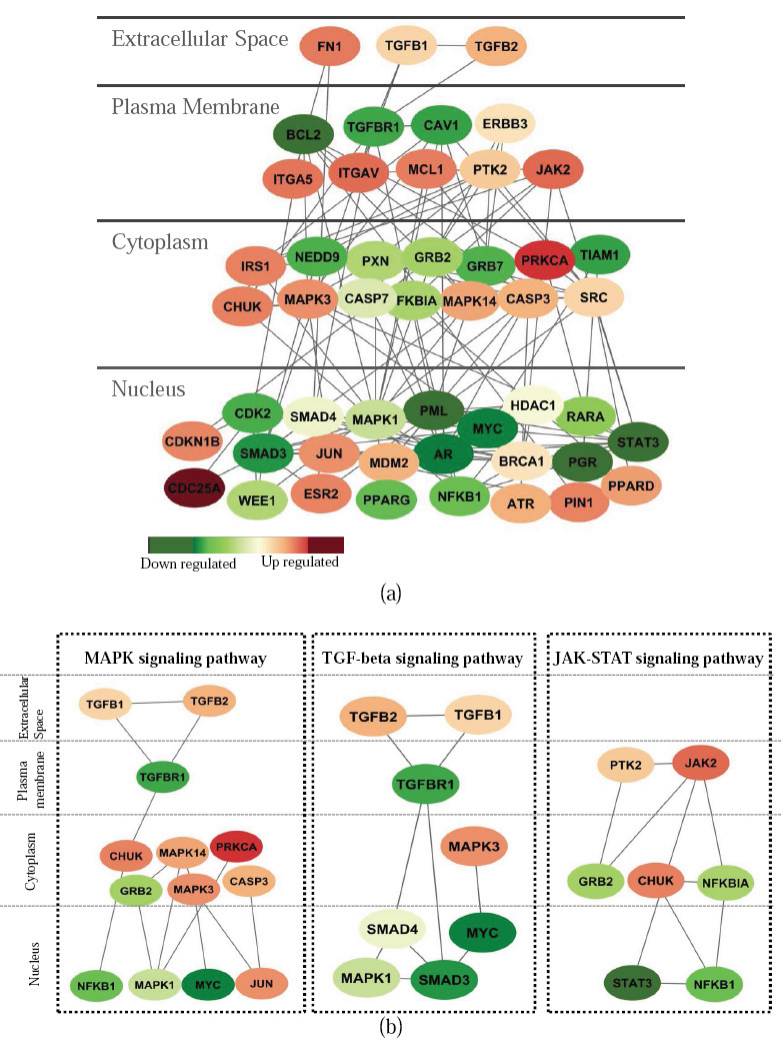
**(a) Subnetworks from the top 50 genes identified by netSVM on Wang *et al***. [24]**; (b) signaling pathways highlighted in the identified subnetworks including MAPK, TGF-beta and JAK-STAT signaling pathways**.

To obtain a more detailed comparison and understanding of the subnetworks identified by SVM and netSVM, we selected the top 50 genes (p-value threshold 0.05) to check the subnetworks from van de Vijver *et al*. [23] and Wang *et al*. [24]. For SVM, 20 genes (17 edges) in van de Vijver *et al*. [23] and 18 genes (15 edges) on Wang *et al*. [24] are connected to form subnetworks. Only 5 genes overlap in the two subnetworks. For netSVM, 47 genes (100 edges) on van de Vijver *et al*. [23] (shown in Figure 11) and 49 genes (131 edges) on Wang *et al*. [24] (shown in Figure 12) are connected to form subnetworks. Moreover, 17 genes overlap in the two subnetworks. We further input these gene lists to the DAVID database [25] for functional annotation and pathway enrichment analysis. 'Pathways in cancer' is highly enriched in two subnetworks identified by netSVM (Benjamini p-value = 2.1 e-12 on van de Vijver *et al*. [23]; Benjamini p-value = 4.6 e-21 on Wang *et al*. [24]), which is much more significant than those obtained with SVM (Benjamini p-value = 0.12 on van de Vijver *et al*. [23]; Benjamini p-value = 1.1 e-6 on Wang *et al*. [24]). The networks are shown in Figures 11 and 12 as displayed by the Cytoscape software [26,27].

Figures 11 and 12 show three major components in each subnetwork and they are quite similar. The first component contains common (or shared) genes of SRC, CHUK, CASP7, HDAC1, MDM2, NFKB1A and JAK2 (the right panels in Figure 11 and Figure 12). The major functional annotations for these genes are apoptosis (p = 5.7 e-7), response to cytokine stimulus (p = 2.4 e-5), chemokine signaling pathway (p = 1.6 e-7) and JAK-STAT signaling pathway (p = 3.4 e-06), as estimated using the DAVID database [25]. The second component includes genes of FN1, CAV1, TGFBR1, MAPK1, MAPK14, MAPK3, SMAD4, and PXN (the left panels in Figure 11 and Figure 12), which are enriched in regulation of apoptosis (p = 8.1 e-7); regulation of growth; regulation of cell proliferation (p = 1.1 e-7); TGF-beta signaling pathway (p = 1.8 e-6) and MAPK signaling pathway (p = 3.1 e-5). For the remaining genes, one is centered by TP53 (Figure 11) and another is centered by AR and BRCA1 (Figure 12) in the nucleus. Both components are enriched in regulation of cell cycle (p = 7.0 e-5).

The significant genes in the subnetworks from the Wang and van de Vijver data sets potentially represent a strong prognostic signature in breast cancer. The functions of most of these genes are related to biological pathways already known to be involved in disease progression, such as apoptosis, cell cycle and cell proliferation, and these functional results are consistent with ones discovered in the original studies [23,24]. Importantly, some gene itself may not show differential expression between two phenotypes, but may play an important role in interconnecting other differentially expressed genes in PPI network [14]. Therefore we consider the genes with high degree of interactions in PPI network as hub genes. Our proposed method can highlight several hub genes and signaling pathways that were not identified in the original studies, such as MAPK, TGF-beta, and JAK-STAT signaling pathways (see Figures 11(b) &12(b)). The subnetworks from two data sets have been extensively studied in Chuang *et al*. [14], where many subnetworks are functionally related to signaling of cell growth and survival, cell proliferation and replication, apoptosis, metabolism, etc. However, with the limitation inherited from a local search, many subnetworks only contain a small number of genes, which makes it difficult to gain a global picture of underlying biological mechanisms. This is especially problematic for signaling pathways, because signaling pathways are considered to be more global (from membrane to cytoplasm and to nucleus) rather than local protein interactions. As a comparison, the networks identified by netSVM are more related to signaling pathways; and the genes in the networks are likely to be associated with diverse cellular locations ranging from extracellular matrix, plasma membrane, cytoplasm and nucleus (Figures 11(b) &12(b)).

In estrogen receptor-positive breast cancer, MAPK activation is robustly increased during ligand (estrogen)-independent cell proliferation resulting from long-term estrogen deprivation [28], and combined inhibition of the Ras/MAPK and Notch signaling pathways is being explored as a potential new modality for breast cancer treatment [29]. A previous study [30] has also shown that MAPK inhibition in estrogen receptor-negative breast cancer cell lines can restore estrogen receptor expression and growth inhibition by the antiestrogen Tamoxifen. Recent studies have further shown that activation of MAPK signaling pathway could mediate response to Tamoxifen in breast cancer patients [31] and the combination of MAPK and PI3K inhibitors is an effective strategy to overcome endocrine therapy resistance [32]. Transforming growth factor-beta (TGF-beta) is often considered a tumor suppressor, which is implicated in many types of human cancer including breast cancer [33]. However, other recent studies have shown that TGF-beta signaling may positively influence the metastatic cascade in breast cancer by enabling cells to become motile and enhancing the ability of cells to survive clearance from the lungs during the metastatic process [34]. Regulation of JAK-STAT signaling is highly complex and involves cross-talk with numerous other signaling pathways. For example, the functions of activated STATs can be altered through association with other transcription factors such as c-Jun, c-Fos, NF-KappaB, SMAD, SP1, p300, CBP, BRCA1 and MCM5 [35]. Furthermore, STAT1 [36], STAT3 [37] and STAT5 [38] have all been shown to play important roles in endocrine-resistant breast cancer.

As a final note, the prediction accuracy from two data sets is not high enough for recurrence prediction of breast cancer for clinical applications. This limitation is a challenge to the field, which is largely caused by the sample heterogeneity, complexity of breast cancer and experimental noise in microarray data. However, our method can achieve a comparable performance with other network-based methods. Besides, our method can identify important network biomarkers that are functionally related to breast cancer, aiming for a mechanistic understanding of breast cancer. The networks and enriched pathways identified from two data sets have shown that there is a convergent point at the functional level even with a large discrepancy observed at the gene expression level.

## Conclusion

In this paper, we have developed a novel method (netSVM) for cancer biomarker identification that incorporates gene-gene interaction information. This network information has been explicitly formulated as a Laplacian matrix and embedded into the objective function of SVM for optimization. Therefore, the contribution of hub genes to the classification hyperplanes of SVM is greatly enhanced, even when these hub genes are not significantly differentially expressed between the two phenotypes. Our method for subnetwork identification in simulated and breast cancer data shows significantly improved reproducibility of prediction performance across different data sets when compared to other network-based methods and gene-based methods. Finally, several signaling pathways revealed by netSVM have high functional relevance to breast cancer, and these may provide us new insight into the underlying mechanism of breast cancer progression and metastasis.

The proposed method works under the assumption that hub genes usually have little expression changes, thus to help improve the generalizability across different data sets by integrating network information. The method may not achieve an improved performance if the assumption is violated. In addition, since the proposed method utilizes protein-protein interaction data as prior knowledge, the performance largely relies on the correctness of prior knowledge. Therefore in future, it is necessary to assess the influence of prior knowledge onto the method. Meanwhile, it is desirable to incorporate more sophisticated network identification approaches into this method to improve the prediction accuracy for clinical applications.

Although we focused on a breast cancer study in the paper, the proposed method can be generalized to different applications (e.g., studying drug resistance in breast cancer) or other cancer studies (e.g., ovarian cancer) to identify biomarkers by integrating expression data and protein-protein interaction network. The proposed method could be further extended to general classification problems when the features are dependent and interact with each other. In such case, netSVM can provide an effective way to impose constraints on the features to model their dependency hence to improve the reproducibility of the classifier.

## Methods

Support vector machine (SVM) is a classification scheme that addresses the general case of nonlinear and non-separable classification tasks efficiently. The goal of an SVM is to find a hyperplane that maximizes the width of the margin between the classes and at the same time minimizes the empirical errors. Since the coefficients in weight vector correspond to real genes for linear SVM, we will focus on discussing the network-constrained SVM for linear case only in order to have a clear biological interpretation of those significant features (i.e., genes).

### Support vector machine

Given a training sample set (**x**_1_, *y*_1_),..., (**x***_l_*, *y_l_*) with *p *features and *l *samples, where **x***_i _*∈ *R^p ^*and *y_i _*∈ {-1 + 1}, the SVM learning algorithm aims to find a linear function of the form *f*(**x**) = **β **· **x **+ *b*, with **β **∈ *R^p ^*and *b *∈ *R *such that a data point **x **is assigned to a label +1 if *f*(**x**) > 0, and a label -1 otherwise. The linear SVM classifier can be obtained by solving the following optimization problem:

(1) $$\begin{array}{c} \underset{\beta ,b,\xi }{\min}\frac{1}{2}||\beta |{|}^{2}+C\overset{l}{\underset{i=1}{\sum }}\xi_{i}\\ s.t.y_{i}\left(\beta \cdot x_{i}+b\right)\geq 1-\xi_{i},\xi_{i}\geq 0 \end{array},$$

where the slack variable *ξ_i _*> 0 denotes the difference of sample *i *to the required functional margin. The sum of *ξ_i _*can be seen as an upper bound of the empirical risk. And the regularization constant *C *> 0 determines the trade-off between 1/2||**β**||² (the complexity term) and the sum of *ξ_i_*.

By introducing non-negative Lagrangian multipliers *α_i_*, the above optimization problem is equivalent to maximizing the dual Lagrangian function with respect to *α_i _*in Equation (2):

(2) $$\begin{array}{c} L_{D}\left(\alpha \right)=\overset{l}{\underset{i=1}{\sum }}\alpha_{i}-\frac{1}{2}\overset{l}{\underset{i,j=1}{\sum }}\alpha_{i}\alpha_{j}y_{i}y_{j}x_{i}\cdot x_{j},\\ s.t.\;\forall i\;0\leq \alpha_{i}\leq C\\ \overset{l}{\underset{i=1}{\sum }}\alpha_{i}y_{i}=0 \end{array}.$$

This is a quadratic programming problem and the solution to Equation (2) gives that $\beta =\overset{l}{\underset{i=1}{\sum }}\alpha_{i}y_{i}x_{i}$, while *b *can be simply computed with any training point such that equality holds in Equation (1).

### Network-constrained SVM

Consider a gene network that is represented by a graph *G *= (*V*, *E*, *W*), where *V *is a set of vertices that correspond to *p *genes, *E *= {*u *~ *v*} is a set of edges indicating that gene *u *and *v *are linked on the network and *W *is the weights of the edges. The degree of a vertex *v *is defined as $d_{v}=\underset{u}{\sum }w\left(u,v\right)$, where *w*(*u*, *v*) indicates the weight of edge *u~v*. For this application, the weights could represent the probabilities of having edges between two vertices. Following Chung *et al*. [39], we define the Laplacian matrix **L **of *G *with the *uv*^th ^element to be:

(3) $$L\left(u,v\right)=\left\{\begin{array}{c} 1-\frac{w\left(u,v\right)}{d_{u}}\\ \frac{-w\left(u,v\right)}{\sqrt{d_{u}d_{v}}}\\ 0 \end{array}\;\begin{array}{c} if\;u=v\;and\;d_{u}\neq 0\\ if\;u\;and\;v\;are\;adjacent.\\ otherwise \end{array}\right)$$

This matrix is symmetric and non-negative definite and its corresponding eigenvalues or spectra reflect many properties of the graph as detailed in [39].

We define the network-constrained SVM given non-negative parameter *λ *as follows:

(4) $$\begin{array}{c} \underset{\beta ,b,\xi }{\min}\frac{1}{2}\beta^{T}\beta +\lambda \beta^{T}L\beta +C\overset{∕}{\underset{i=1}{\sum }}\xi_{i}\\ s.t.y_{i}\left(\beta \cdot x_{j}+b\right)\geq 1-\xi_{i},\xi_{i}\geq 0 \end{array}.$$

Compared to Equation (1), the only difference is that we add one more regularization term *λ***β***^T^***Lβ **into the objective function. We already know that the first regularization term is designed to maximize the width of the margin between two classes. We will thus focus on discussing the meaning of the second regularization term.

Note that **L **can be written as **L **= **SS***^T^*, where **S **is the matrix whose rows are indexed by the vertices and whose columns are indexed by the edges of G such that each column (corresponding to an edge e = {*u*, *v*}) has an entry $\sqrt{w\left(u,v\right)}∕\sqrt{d_{u}}$ in the row corresponding to *u*, an entry $-\sqrt{w\left(u,v\right)}∕\sqrt{d_{u}}$ in the row corresponding to *v*, and zero entries elsewhere. Therefore we can see that **β***^T^***Lβ **can be re-written as

(5) $$\beta^{T}L\beta =\underset{u-v}{\sum }{\left(\frac{\beta_{u}}{\sqrt{d_{u}}}-\frac{\beta_{v}}{\sqrt{d_{v}}}\right)}^{2}w\left(u,v\right).$$

From this representation we can understand that the added regularization term *λ***β***^T^***Lβ **imposes the smoothness of parameters (coefficients) **β **over the network via penalizing the weighted sum of squares of the scaled difference of coefficients between neighboring vertices in the network.

It is worth noting that the network-constrain SVM is different from Laplacian SVM [40]. Network-constrained SVM imposes smoothness for weight vector **β**, while Laplacian SVM imposes smoothness for Lagrangian multipliers *α*. In Laplacian SVM, it assumes that the data of each class, which follow a manifold and decision function must avoid passing through the manifold. In network-constrained SVM, the underlying assumption is that the genes highly connected in a network have synergistic effect and they should be considered together rather than individually.

Next, we will discuss how to solve the problem of Equation (4). Here we propose a simple algorithm by reducing it to a conventional SVM optimization problem. Since **L **is symmetric and semi-positive definite, Equation (4) can be represented as

(6) $$\begin{array}{c} \underset{\beta ,b,\xi }{\min}\frac{1}{2}\beta^{T}L^{*}\beta +C\overset{l}{\underset{i=1}{\sum }}\xi_{i}\\ s.t.y_{i}\left(\beta \cdot x_{i}+b\right)\geq 1-\xi_{i},\xi_{i}\geq 0 \end{array},$$

where,

(7) $$\begin{array}{c} L^{*}=\left(I+2\lambda L\right)=U\Gamma {\text{U}}^{T}\\ \;=U\Gamma^{1∕2}\Gamma^{1∕2}U^{T}=PP^{T}.\\ \;\text{when }P=U\Gamma^{1∕2} \end{array}$$

Further with the definition of β* = **P***^T^***β**, the problem in Equation (6) can be reduced to

(8) $$\begin{array}{c} \underset{\beta^{*},b,\xi }{\min}\frac{1}{2}\beta^{*T}\beta^{*}+C\overset{l}{\underset{i=1}{\sum }}\xi_{i}\\ s.t.y_{i}\left(\beta^{*}\cdot x_{i}^{*}+b\right)\geq 1-\xi_{i},\xi_{i}\geq 0 \end{array},$$

where $x_{i}^{*}={\left({\left(P^{T}\right)}^{-1}\right)}^{T}x_{i}$. Therefore, this optimization problem can be solved by its corresponding dual problem similar to Equation (2). The solution gives that $\beta^{*}=\overset{l}{\underset{i=1}{\sum }}\alpha_{i}y_{i}x_{i}^{*}$ and we can recover **β **through **β **= (**P***^T^*)^-1^β*. Note that *λ *is a parameter that can be optimized through cross validation in practice.

### Significance analysis of subnetworks defined by netSVM

From the input network, we want to know which parts of the network are significantly contributing to the decision boundary for classification. As is shown in the Equation (4), the larger the absolute value of an element in coefficient vector **β**, the more important the corresponding gene is. Based on the clinical outcome information, we design a significance test to evaluate the significance of each gene in the network and then significant subnetworks can be determined by those genes whose p-values are less than some predefined threshold. For each gene *i *in the network, we take its absolute value of coefficient *β_i _*as a summary statistic. To form a null distribution, we randomly permute training sample labels, and learn the coefficient vector **β**^0 ^using network-constrained SVM on the training samples with permuted labels. The procedure is repeated *B *times, and all the corresponding absolute values of *β_i_*^0 ^will be used to form the null distribution. The p-value of gene *i *can be calculated as follows:

(9) $$\begin{array}{c} p_{i}=\text{P}{\text{r}}_{H_{0}}\left(\left|\beta_{i}^{0}|\;>|\beta_{i}\right|\right)\\ \;\;=\frac{\#\left\{b:|\beta_{i}^{0b}|\;>|\beta_{i}|,b=1,\cdot \cdot \cdot B\right\}.}{B} \end{array}$$

### Simulation of microarray gene expression data

We modified a Markov random field (MRF) model in [17] to embed differentially expressed subnetwork/genes in a PPI network given a ground truth subnetwork. Let *S *be a binary vector indicating the differential expressed states of genes in a PPI network G, 0 representing 'equally expressed' ('EE')and 1 representing 'differentially expressed' ('DE'). Assume the ground truth differential subnetwork is G_0_, which means *S*_{G0} _= 1 and *S*_{G-G0} _= 0. We sample the gene state according to the following probability based on Markov random field model:

(10) $$p_{i}\left(k|\cdot \right)\propto \exp\left(\gamma_{k}-\chi \mu_{i}\left(1-k\right)\right).$$

In the original model, *μ_i_*(1-*k*) denotes the number of neighbors of gene *i *having state 1-*k*, *k *= 0, 1. *γ_k _*and *χ *are the parameters predefined. In order to introduce different level of false positives in the sampled differential subnetwork, we added one parameter to control the probability of keeping initial states of ground truth DE genes and background EE genes. Here we define *μ_i_*(1-*k*) as a function of parameter *ω *as follows:

(11) $$\begin{array}{c} \mu_{i}\left(1-k\right)=\frac{\omega \cdot \left(1-S_{i}^{1-k}\right)+\underset{j\in N_{l}}{\sum }\left(1-S_{j}^{1-k}\right)}{\omega +\underset{j\in N_{l}}{\sum }\left(S_{j}^{1-k}+S_{j}^{k}\right)},\\ \text{where }\;S^{1}=S,\;S^{0}=1-S. \end{array}$$

The larger *ω is*, the more consistent the simulated DE genes and ground truth genes are. Therefore we can vary *ω *to generate different simulation gene expression data sets with different levels of consistency.

Then, we simulated gene expression data *X *given *S *using a Gamma-Gamma (GG) model [18,41]. In the GG model, the observed variable *x *(gene expression level) is a Gamma distribution having shape parameter *α*>0 and scale parameter *χ_g_*, with a mean value *μ_g _*= *αχ*_*g*_. Its probability density function is:

(12) p(x|α,χg)=xα-1 exp{-x∕χg}χgαΓ(α).

In the above equation, the scale parameter *χ_g _*has a Gamma distribution with shape parameter *α_0 _*and scale parameter *ν*. Given these three parameters, we can simulate gene expression levels in two conditions with multiple replicates. Particularly for this study, we assume that equally expressed gene has same expected mean value for all samples and differentially expressed gene has different expected mean values for samples in different conditions. We fist sampled the scale parameter *χ_g _*based on Gamma distribution (*α_0_, ν*) and then sampled gene expression levels using parameters (*α, χ_g_*) given the states of genes.

## Authors' contributions

LC and JX designed the framework of the proposed method. LC constructed and implemented the method and performed simulation experiments. LC and JX designed the breast cancer study and LC performed the data analysis. RR and RC provided their biological interpretation on the breast cancer results. LC and JX wrote and revised the manuscript with the help from RR, RC and YW. All authors read and approved the final manuscript.
